# Supplementary material for: A Novel R2R3-MYB Transcription Factor SbMYB12 Positively Regulates Baicalin Biosynthesis in Scutellaria baicalensis Georgi
Source: Int J Mol Sci. 2022 Dec 7;23(24):15452. doi: 10.3390/ijms232415452 (PMC9778813; doi:10.3390/ijms232415452)
Supplement: Supplementary file 1 [file ijms-23-15452-s001.zip › Supplementary Material Legends.docx]

**Supplementary Tables:**

**Table S1.** List of primers used in the study

**Table S2.** Amino acids sequence of *Sb*MYB12 and other MYBs from other species

**Supplementary Figures:**

**Figure S1**. *SbMYB12* Nucleotide sequence and deduced amino acid sequences. Deduced amino acid sequences are displayed beneath nucleotide sequences, and the asterisk indicates the stop code. Underlined are the conserved R2 and R3 domains.

**Figure S2**. *Sb*MYB12 protein bioinformatics analyses. (**A**) Analyses of hydrophilicity and hydrophobicity. (**B**) analysis of transmembrane domains. (**C**) analysis of signaling peptides.

**Figure S3**. Procedures for generating transgenic *S. baicalensis* hairy roots. (**A**) Aseptic *S. baicalensis* plant (**B**) Pre-cultivation and co-cultivation of stems. (**C**) Cultivation of stems on the selected medium B5 (Cef+Kana). (**D**) Hairy roots after excision from stems. (**E**) Hairy roots were cultured in a B5 liquid medium.
