# Supplementary figures and images for: A Novel R2R3-MYB Transcription Factor SbMYB12 Positively Regulates Baicalin Biosynthesis in Scutellaria baicalensis Georgi
Source: Int J Mol Sci. 2022 Dec 7;23(24):15452. doi: 10.3390/ijms232415452 (PMC9778813; doi:10.3390/ijms232415452)

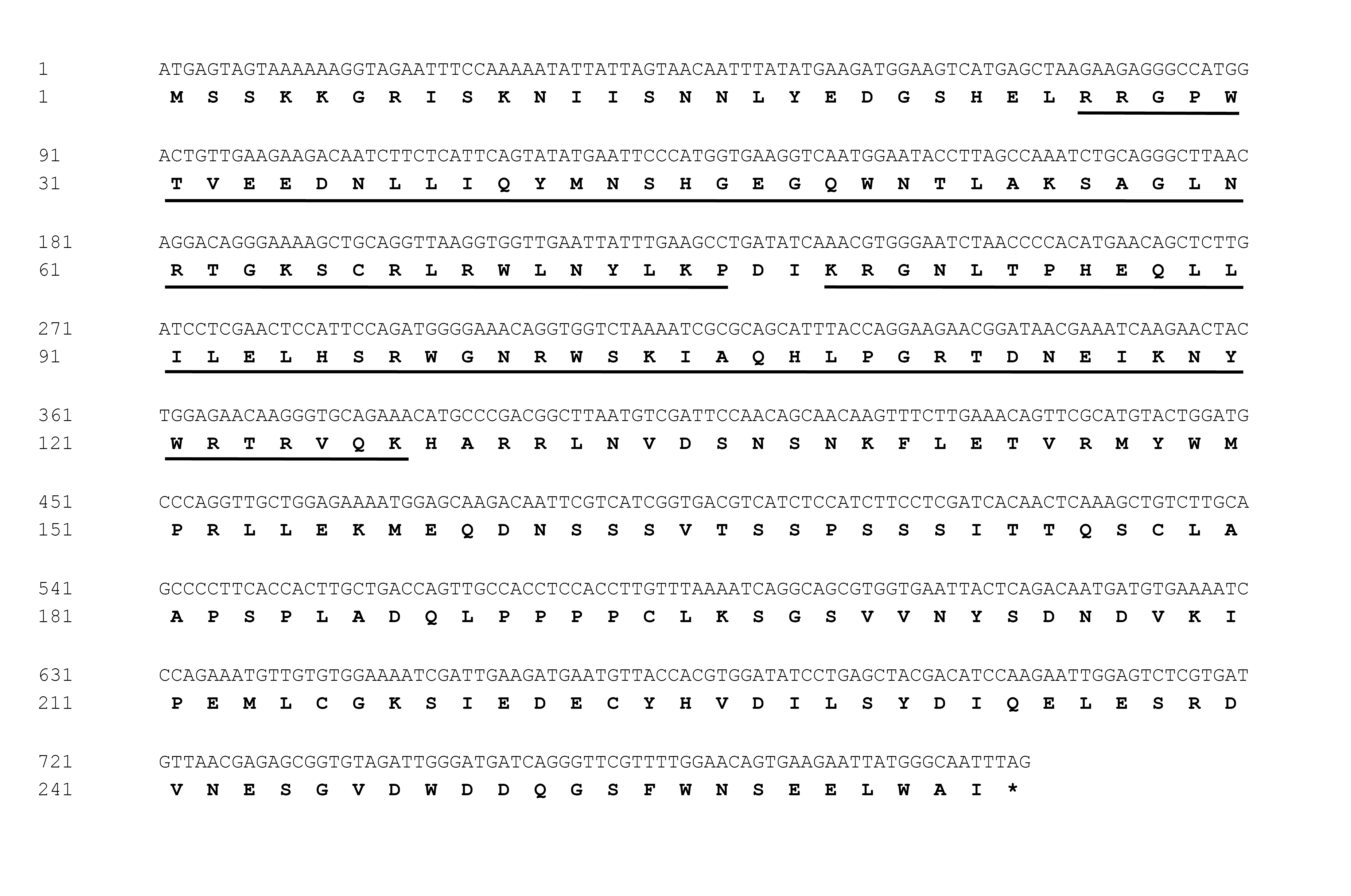

Supplement: Supplementary file 1 [file ijms-23-15452-s001.zip › Figure S1.tif]

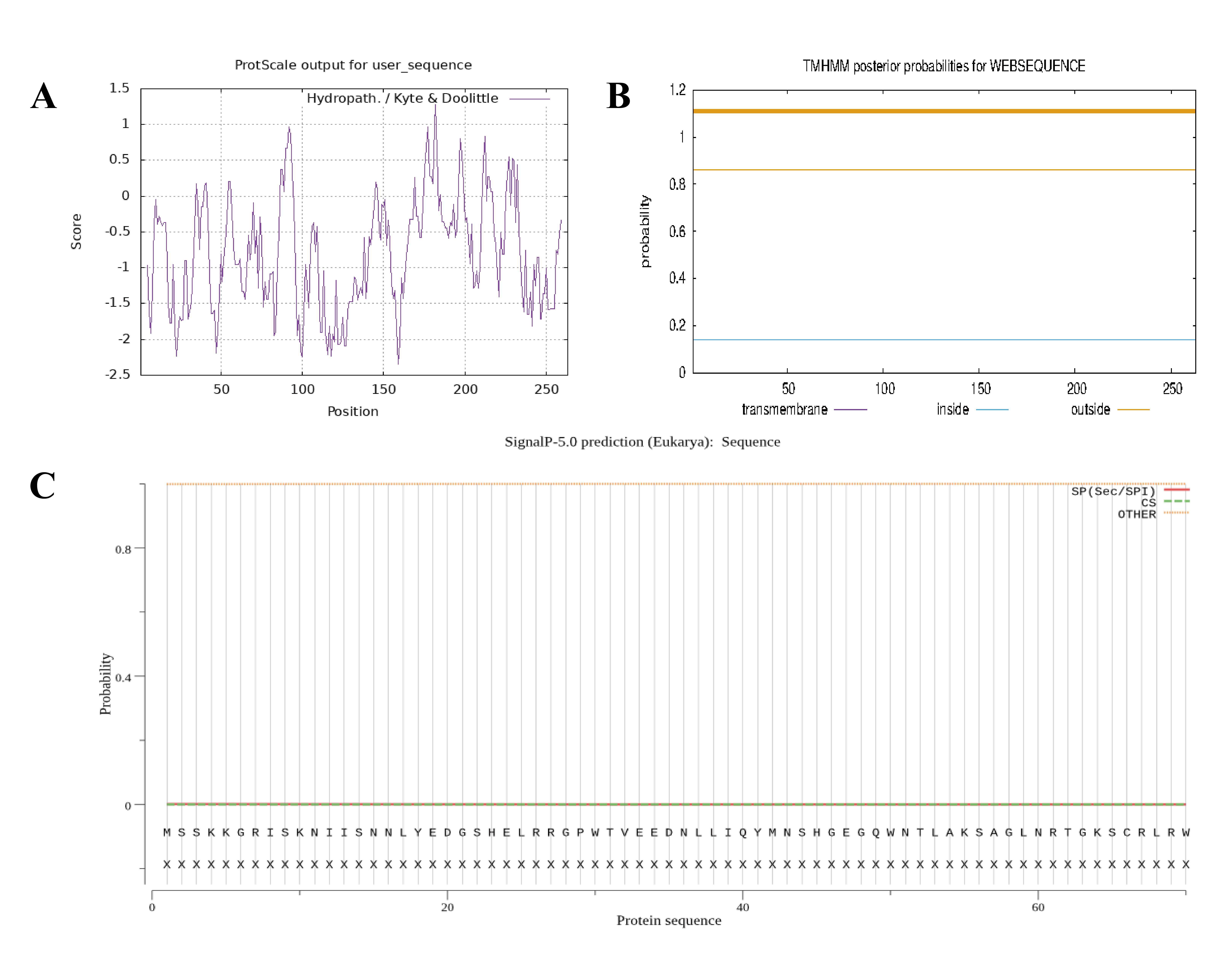

Supplement: Supplementary file 1 [file ijms-23-15452-s001.zip › Figure S2.tif]

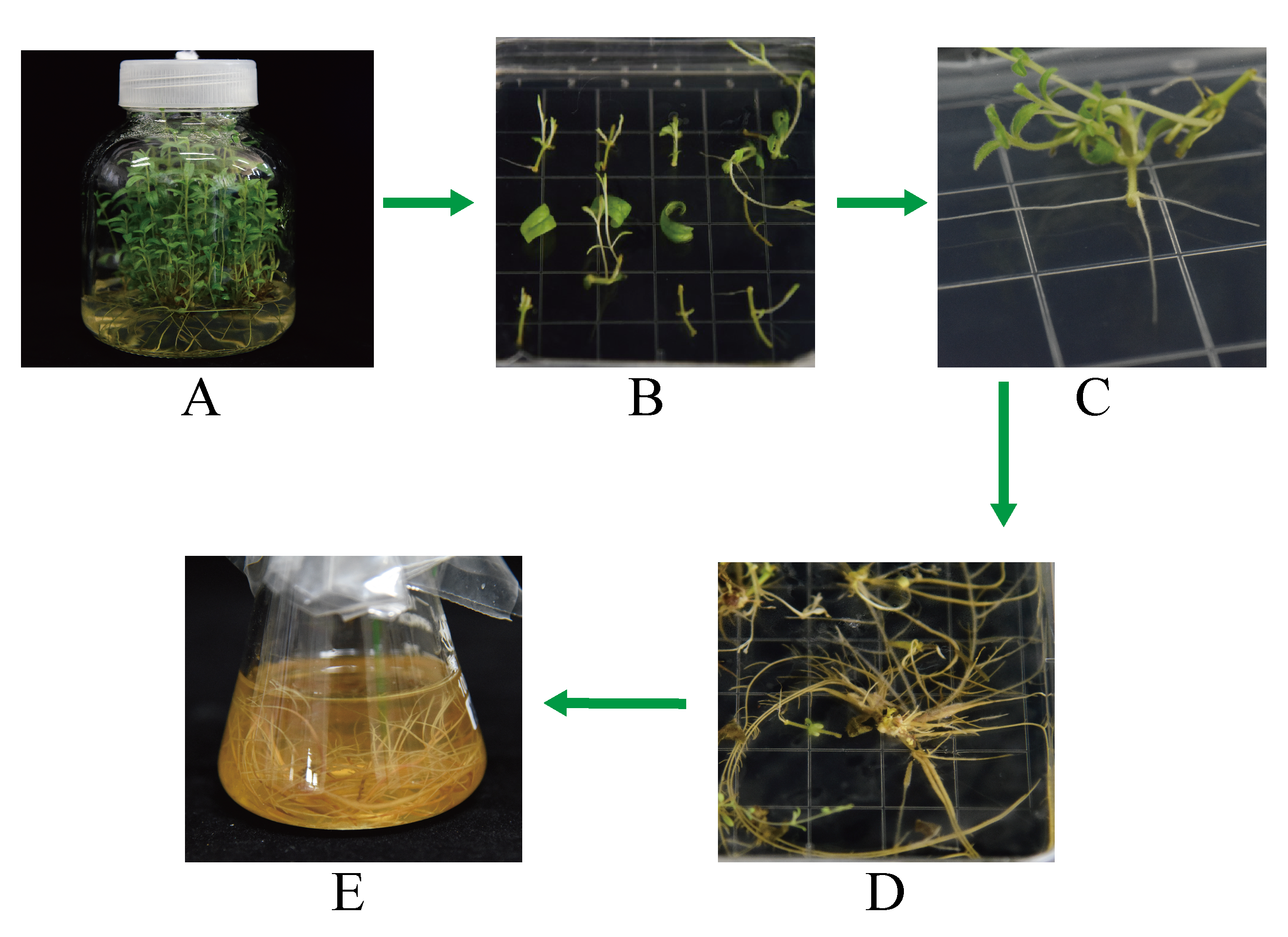

Supplement: Supplementary file 1 [file ijms-23-15452-s001.zip › Figure S3.tif]
